# Performance assessment and process optimization of a sulfur recovery unit: a real starting up plant

**DOI:** 10.1007/s10661-023-10955-x

**Published:** 2023-02-03

**Authors:** Ahmed Y. Ibrahim, Fatma H. Ashour, Mamdouh A. Gadalla, Amal Abdelhaleem

**Affiliations:** 1 Department of Chemical Engineering, Cairo University, Giza, 12613 Egypt; 2 Department of Chemical Engineering, Port Said University, 42526 Port Fouad, Egypt; 3 Department of Chemical Engineering, The British University in Egypt, Misr-Ismalia Road, El-Shorouk City, 11837 Cairo, Egypt; 4 Environmental Engineering Department, Egypt-Japan University of Science and Technology, New Borg El-Arab City, Alexandria, 21934 Egypt

**Keywords:** Sulfur recovery unit, Optimization, Environment, Performance test guarantee

## Abstract

Sulfur recovery units (SRU) have an important role in the industrial production of elemental sulfur from hydrogen sulfide, whereas the generated acidic gas emissions must be controlled and treated based on local and international environmental regulations. Herein, Aspen HYSYS V.11 with Sulsim software is used to simulate the industrial and treatment processes in a refinery plant in the Middle East. In the simulation models, in temperature, pressure, flow, energy, and gas emissions were monitored to predict any expected change that could occur during the industrial processes. The simulation models were validated by comparing the obtained data with actual industrial data, and the results showed low deviation values. The simulation results showed that the current process temperature conditions can work efficiently for sulfur production without causing any environmental consequences. Interestingly, the simulation results revealed that sulfur can be produced under the optimized temperature conditions (20° less than design temperatures) with a total amount of steam reduction by 1040.12 kg/h and without any negative impact on the environment. The steam reduction could have a great economic return, where an average cost of 7.6 $ per ton could be saved with a total estimated cost savings by 69,247.03 $ per year. The simulation revealed an inaccurate production capacity calculated by real data in the plant during the performance test guarantee (PTG) where the real data achieved around 1 ton/h higher capacity than the simulation result, with an overall recovery efficiency of 99.96%. Based on this significant result, a solution was raised, and the level transmitters were calibrated, then the test was repeated. The simulation models could be very useful for engineers to investigate and optimize the reaction conditions during the industrial process in sulfur production facilities. Hence, the engineers can utilize these models to recognize any potential problem, thereby providing effective and fast solutions. Additionally, the simulation models could participate in assessing the performance test guarantee (PTG) calculations provided by the contractor.

## Introduction

Hydrogen sulfide produced in the refining industry is regarded as a hazardous pollutant due to its toxic and acidic nature (Khatami et al., 2016). In order to comply with international environmental regulations, sulfur recovery unit (SRU) plants convert hydrogen sulfide into elemental sulfur (Lavery et al., 2019; Mahmoodi et al., 2017) and stop the emission of any acidic gases (Ibrahim et al., 2017; Abdoli et al., 2019; Sui et al., 2019).

Claus process is one of the oldest, common, and useful method to do this role (Hosseini et al., 2019). Several methods have been developed for increasing SRU recovery (Rostami et al., 2019). Most of the plants use the modified Claus process to produce sulfur. The common principle of the process is to divide hydrogen sulfide feed into two parts, in the first part, one-third of acidic gas feed forms SO_2_; SO_2_ reacts with the remaining two-thirds of H_2_S to form sulfur (Kazempour et al., 2017).

The SRU is mainly divided into two sections: thermal and catalytic. The thermal section consists of a waste heat boiler (WHB) for heat recovery and a thermal reactor Claus furnace. One-third of the H_2_S is oxidized in the thermal reactor via reaction (Eq. 1), and the remaining two-thirds of the H_2_S are then reacted with the SO2 generated in the thermal reactor to produce sulfur in the catalytic section (Eq. 2).

The hot flue gas from the Claus furnace, which also contains COS and CS_2_ by-products, is cooled in the WHB by a water stream to create high pressure steam. After cooling in the sulfur condenser, elemental sulfur is recovered. The Claus furnace typically converts (55–65%) H_2_S. In order to perform the Claus reaction, which produces sulfur, and the hydrolysis reactions, which convert COS and CS_2_ to H_2_S, the process gas exiting the thermal section is reheated to an appropriate temperature. In order to prevent sulfur condensation, the temperature is raised above the dew point of sulfur. After passing through the first catalytic reactor, sulfur is produced by the Claus reaction (Eq. 2). Through the reactions (Eqs. 3 and 4), the first catalytic reactor also carries out COS and CS_2_ hydrolysis.

1 $$\mathrm{H}_{2}\mathrm{S} + 1.5\mathrm{O} \ 2 \to \mathrm{SO}_{2} + \mathrm{H}_{2}\mathrm{O}$$

2 $$2\mathrm{H}_{2}\mathrm{S} + \mathrm{SO}_{2} \to 3/8\mathrm{S}_{8} + 2 \ \mathrm{H}_{2}\mathrm{O}$$

3 $$\mathrm{CS}_{2} + 2\mathrm{H}_{2}\mathrm{O} \rightleftharpoons \mathrm{CO}_{2} + 2\mathrm{H}_{2}\mathrm{S}$$

4 $$\mathrm{COS} + \mathrm{H}_{2}\mathrm{O} \rightleftharpoons \mathrm{CO}_{2} + \mathrm{H}_{2}\mathrm{S}$$

5 $$2\mathrm{NH}_{3} + 1.5 \ \mathrm{O}_{2}\to \mathrm{N}_{2} + 3 \ \mathrm{H}_{2}\mathrm{O}$$

A catalytic unit consists of a reheater prior to the catalytic reactor and a condenser following the reactor. The 2-stage’s maximum overall sulfur recovery efficiency (SRE), which includes the thermal and catalytic sections, is 93–95%. The 3-stage catalytic units have an SRE of 96–98%. The addition of a tail gas treatment unit (TGTU) to the modified Claus process can help achieve the 99.9% SRE needed to comply with environmental regulations in recent years (Ibrahim, 2021a, b; Mehmood et al., 2020; Ibrahim et al., 2021a, b, c, d, e, f; Sui et al., 2019; Ghahraloud et al., 2017).

Some hazardous contaminants are present in process sour water produced by refinery plants. The main pollutants in sour water are H_2_S and ammonia (Dardor et al., 2019; Minier-Matar et al., 2017; Gai et al., 2020). Strippers are used to remove H_2_S and NH_3_ from contaminated water (Hassan-Beck et al., 2019; Zahid, 2019; Zhu et al., 2016).

Amine treating units are used to sweeten sour gas that contains acid gas such as H_2_S. The gas is exposed to a lean amine solution, which absorbs H_2_S, and the H_2_S is then stripped from the rich amine in a regenerator (Wang et al., 2019; Amini et al., 2018). Diethanolamine (DEA) and methyldiethanolamine (MDEA) amines are commonly used to perform this function (Aghel et al., 2019; Mohamadi-Baghmoleaei et al., 2020; Abdolahi-Mansoorkhani & Seddighi, 2019; Pashaei & Ghaemi, 2020). When an acidic gas contains both CO_2_ and H_2_S, MDEA is used because of its high selectivity to H_2_S over CO2 (Pal et al., 2015; Concepción et al., 2020, Shunji et al., 2020).

The SRU plant is fed by H_2_S and NH_3_ produced by sour water stripping (SWS) and amine regeneration units (ARU) (Ibrahim, 2021a, b).

Process modeling and simulations are crucial methods for investigating and assessing the efficiency and predictability of existing plants, and it is beneficial when developing new plants (Rahman et al., 2019). To improve the performance of the sulfur recovery unit in the Claus process, models such as thermodynamic and kinetic ones have been developed. For instance, several modeling approaches such as genetic algorithms, model-based optimization, modeling, and multi-optimization have been applied (Rao & Haydary, 2019). Exergy investigations have become a hot topic of some recent studies. Energy is permanently destroyed as it is changed from one form to another. Hence, exergy analysis examines the energy lost during a process; sometimes, machinery with high energy efficiency also has substantial energy loss (Zarei, 2020; Hashemi et al., 2019).

Rostami and Tavan; Hashemi et al.; and Zarei conducted exergy research on SRU plants that considered the exergy of the SRU as a whole, the differences between particular sections and exergy studies on specific pieces of equipment (Rostami & Tavan, 2019; Zarei, 2020; Hashemi et al., 2019). Additionally, two exergy analyses were carried out by Ibrahim et al. for two amine regeneration units in the same refinery (Ibrahim, 2021a, b; Ibrahim et al., 2021a, b, c, d, e, f). In the SRU facility, they also performed an exergy assessment for an MDEA scrubber.

**Fig. 1:**
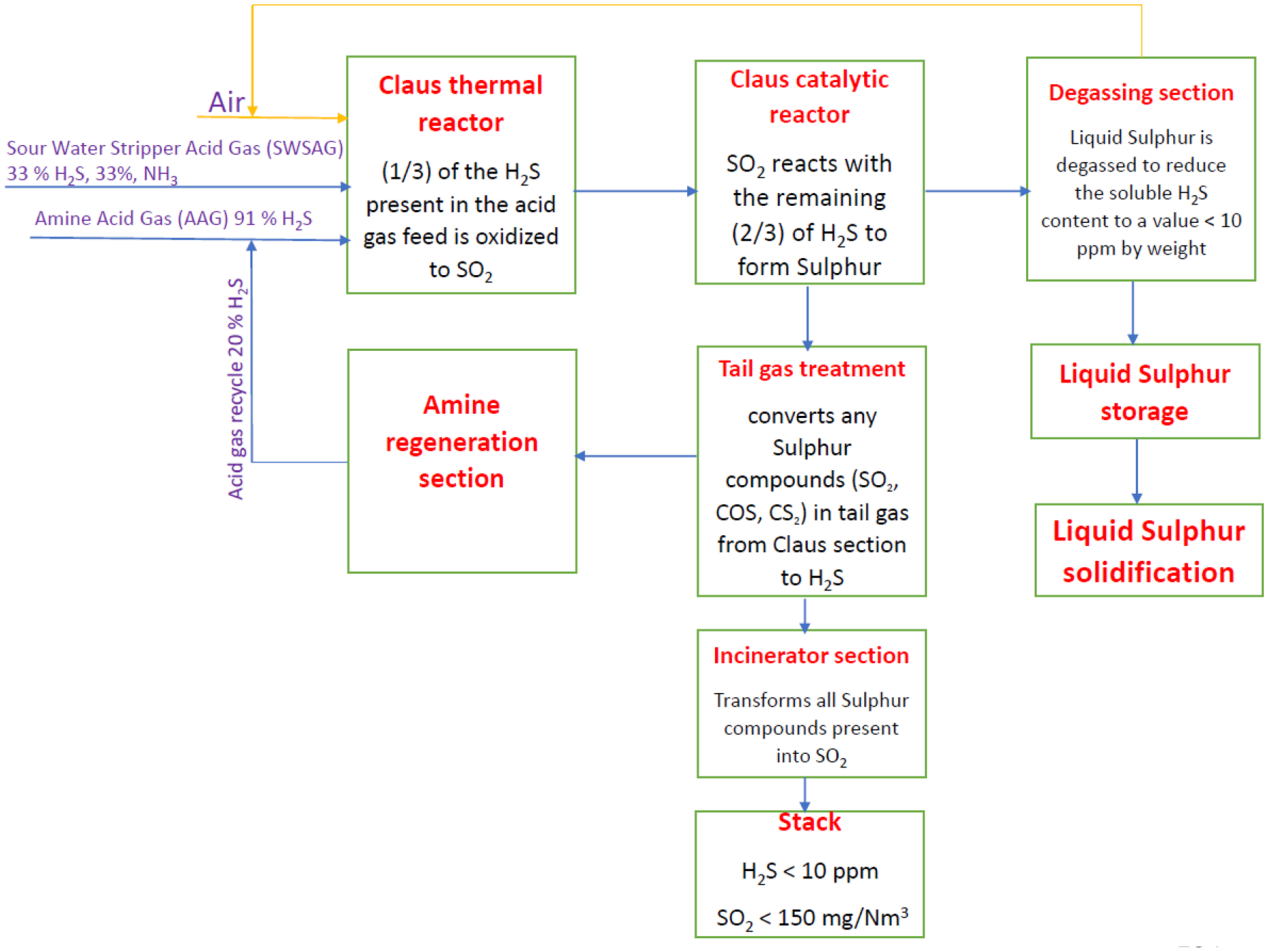
A representation for the main processes in the SRU plant

HYSYS V.11 with Sulsim software is used to simulate a refinery plant in the Middle East that will begin official production in 2020 and use a modified Claus process for treating the plant’s acid gases. This allows researchers to examine real-world issues that arose during the test period in order to solve and optimize them as well as to verify the contractor’s calculations for the performance test guarantee. A representation for the main processes in the SRU plant is shown in Fig. 1. Literature survey found that researchers concentrated on modeling and simulation by other programs like (Promax, Matlab and CFD) but only few studies simulated complete SRU plants using the SRU Sulsim package. The Sulsim (Sulfur Recovery) property package incorporates properties developed by sulfur experts for the purpose of simulating the modified-Claus process and uses the same Gibbs free energy, enthalpy, and viscosity correlations.

The simulation is compared with industrial data for validation and shows accurate results, then study performed on actual problems. During the plant test, it was observed that the inlet process site design temperatures in three exchangers using steam as heating media cannot be achieved.

The steam valves were opened 100% without achieving the process design temperatures, the decision was to check the availability of modifying the equipment as changing the sizing of these valves or to study the availability to work with current and optimized conditions. The changes of valves or equipment in sulfur recovery unit require the shutdown of the unit and consequently the shutdown of all refinery units as it is the last unit receiving H_2_S from the whole refinery. In economic point of view, a shutdown to modify equipment will stop the production of strategic compounds for local usage and export, but on the other hand, decreasing these process temperatures can affect easily sulfur recovery and may lead to acid gas emissions to environmental. Authors before performing this study was believing that current plant conditions will affect the environment. After that, the results surprised the authors themselves because the preheating of combustion air of reaction furnace is a result of other researches to guarantee flame temperature and prevent byproducts formation.

## Problem statement

The plant encountered actual problems in three steam valves, heating different streams of the process; the combustion air inlet to the reaction furnace is heated by steam through a heat exchanger to achieve 240 °C; the steam valve is opened 100% without achieving the desired temperature; and the combustion air is the source of oxygen required for reaction furnace reactions (Eq. 1) (Ibrahim, 2021a, b), (Eq. 5) (Monnery et al., 2001) and oxidation of hydrocarbons (Ibrahim, 2021a, b). This decrease may have a negative impact on decreasing the reaction furnace temperature (Ibrahim et al., 2017), resulting in ammonia not being destructed in the reaction furnace, causing severe problems in the process (Ibrahim, 2021a, b).

The steam valve is opened 100% without heating the process side to achieve the desired temperature; it only achieves around 235 °C; catalytic reactor1 has two roles, the first is sulfur conversion by Claus reaction (Eq. 2) [10] and the second is the hydrolysis reactions of by-products COS and CS_2_ (Eqs. 3 and 4) (Ibrahim, 2021a; Mehmood et al., 2020; Ibrahim, 2021b). The Claus reaction is unaffected by decreasing catalytic reactor temperature1, whereas hydrolysis reactions may be affected, resulting in undesirable outlet by-products from COS and CS_2_ (Khatami et al., 2016). In violation of environmental regulations, COS and CS_2_ may pass through the incinerator to the stack.

The degassing column air inlet is heated by steam via a heat exchanger to 135 °C, and the steam valve is opened completely without achieving the desired process temperature of 127 °C. Air degasses H^2^S traces from sulfur product. This may have an impact on the H_2_S removal efficiency of the sulfur product. Because the SRU is the last unit to receive and treat all acid gases from all refinery units, any change in the parameters that cause sulfur emissions to the stack may result in the shutdown of the sulfur recovery unit, and thus the total refinery shutdown.

## Materials and methods

**Fig. 2:**
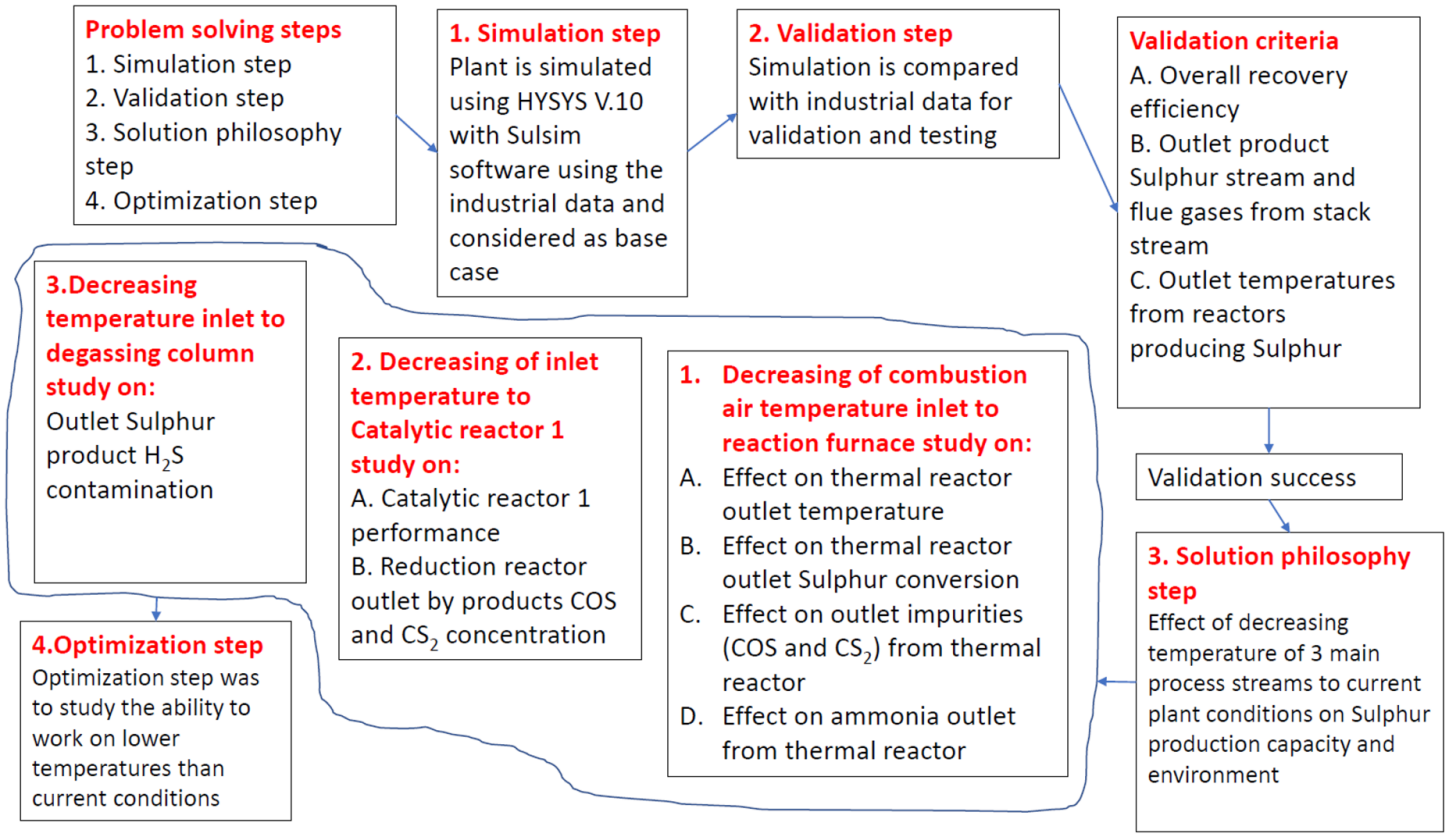
Scheme for methodology

The study used Aspen HYSYS V.11 Sulfur Sulsim package for plant simulation. The problem solving is divided into four steps: simulation, validation, solution philosophy, and optimization. Figure 2 represents a scheme for methodology solution philosophy and optimization steps. The followings are the steps explaining the problem-solving philosophy.

### Simulation step [13, 14]

The entire plant is simulated using Aspen HYSYS V.11 and Sulsim software with plant industrial data as the base case.

#### Simulation sections

The simulation is divided into four sections: Claus, tail gas treatment, degasser, and incinerator. The Claus section is made up of a reaction furnace thermal reactor that converts around 70% of the sulfur product and a waste heat boiler, a catalytic section made up of two catalytic reactors that convert the remaining 30% of the sulfur product and three sulfur condensers, and liquid sulfur is degassed by air to keep the H_2_S content at a safe level of 10 ppm by weight. A reduction reactor, a quench tower, an absorber, and a regeneration section comprise the tail gas treatment section [TGT]. TGT treats Claus tail gas from the catalytic section in order to convert any sulfur compounds into H2S via the reaction of sulfur compounds with hydrogen in the reduction reactor. The converted H_2_S is cooled and absorbed by lean amine before being recycled to the thermal reactor feed for reprocessing. To convert all sulfur compounds present into SO_2_, the tail gas produced by the Claus and TGT units must be incinerated. A stack discharges the incinerated outlet gas to the atmosphere.

#### Simulation criteria

The simulation’s fluid package is Sulsim (Sulfur Recovery), components are chosen from the properties environment components list tab, and the simulation is built in the simulation environment. The main inlet streams are defined as amine acid gas feed (AAG), sour water stripped acid gas (SWSAG), and combustion air to reaction furnace. Stream (mass flow, composition, temperature, and pressure) information is required. HYSYS is normally used to calculate the outlet streams of equipment such as the reaction furnace. More information inlet causes consistency error, which means that the calculation in streams or equipment comes from two different directions, preventing simulation solution. The simulation’s equipment selection was appropriate for the modified Claus process, which included a tail gas treatment section, a degasser section, and an incineration section. Because the Sulsim package can be used for a variety of sulfur technologies, it is critical to correctly select and simulate the appropriate equipment in the package. Incorrect selection leads to a completely different result.

#### Simulation and process description of the Claus section

The Claus section is split into two stages: thermal Claus and catalyst Claus. Typically, the Claus process is used on acidic streams of H_2_S and NH_3_ gases. During the thermal Claus stage, one-third of the H2S present in the acid gas supply is converted into SO_2_, accounting for approximately 70% of the sulfur conversion. The catalyst Claus stage then completes 30% of the sulfur conversion by reacting the remaining H2S with the SO_2_. Following that, the thermal reactor in the Claus stage is fed by the SWSAG and AAG in the presence of sufficient air in the thermal reactor’s main burner to achieve complete oxidation of all hydrocarbons and NH_3_ and buring any remaining H_2_S. In the thermal reactor, exothermic processes occur, and waste heat is recovered by producing high-pressure steam in the WHB. The thermal reactor was described as a “reaction furnace with two chambers” in the SRU package.

Because the feed to the SRU plant contains both gas components, the empirical model used was the “NH_3_ SWSAG” legacy. A single-pass WHB was chosen based on the SRU model package. The two reactors used in the catalytic stage for sulfur conversion were designed as catalytic converters. For the condensation of the generated sulfur, sulfur condensers were used.

#### Simulation and process description of the tail gas treatment section (TGT)

The Claus tail gas (TG) from the Claus stage is treated by the TGTU for SO_2_ to H_2_S conversion. Cooling, absorption by the LA, and recycling to the thermal Claus stage are all used to reprocess the transformed H_2_S. To pre-heat the tail gases leaving the Claus stage, the TG heater heat exchanger uses superheated high-pressure steam as a heating medium. A hydrogen-rich gas stream is combined with the process gas downstream of the heater to provide the necessary hydrogen for the hydrogenation of the sulfur species. A reduction/hydrolysis catalyst is used in the reduction reactor to convert any SO_2_ compounds into H_2_S.

As a result of the exothermic processes, the temperature of the process gas rises, and LP steam is produced via heat recovery in the TGT WHB. The process gas is finally cooled in the quench tower. To complete the H_2_S absorption step, a 45 Wt% concentration of MDEA-based LA solution is used. RA pumps transport the RA solution from the absorber’s bottom to the TGT regeneration section, where it is recycled. After cooling in the LA/RA exchanger and pumping through LA pumps to the LA cooler, the regenerated LA is routed back to the TGT absorber.

The Claus furnace receives any additional feedstock in the form of the acid gas stream that was removed from the RA solution. The reduction reactor is chosen from the SULSIM as a “hydrogenation bed.” Additionally, the amine scrubber unit is significant for amine regeneration and absorption.

#### Simulation and process description of the degassing section

H_2_S and H_2_S_x_ (hydrogen polysulfides) are soluble compounds in the SRU’s liquid sulfur. The presence of H_2_S in the liquid has a negative impact on both safety and the environment due to its toxicity and explosion risk. As a result, liquid sulfur is degassed to reduce the H_2_S level to a safe level of 10 ppm-Wt. The Sulsim package’s “Sulfur degasser” was selected, and the outlet liquid H_2_S concentration was set to 10 ppm-Wt.

#### Simulation and process description of incinerator section

To convert all of the sulfur compounds present in the TG produced by the Claus and TGT units into SO_2_, incineration is required. A stack is used to vent the flue gas produced during incineration into the atmosphere. Because of the extremely low concentrations of its fuel components, the TG ignition temperature is significantly higher than the actual tail gas temperature. As a result, in order to sustain TG combustion, natural gas combustion is required. The incinerator combustion chamber temperature was 650 °C during normal operation, which is required to ensure that the H_2_S and other sulfur compounds present in the TG are completely burned (less than 10 ppm residual H_2_S is anticipated). Because the incinerator has the same name in the HYSYS, the term “incinerator” was used.

### Validation step

The SRU’s goal is to generate sulfur from AG while preventing AG discharge through the stack. As a result, the liquid sulfur product and the flue gas to stack streams were chosen as the two validation streams.

### Solution philosophy

Solution philosophy is an important stage in determining the plant’s ability to function under current conditions. Three process streams’ temperatures are unable to reach design levels. The first affected process stream is the combustion air temperature inlet to the reaction furnace, the second is the temperature inlet to the catalytic reactor1, and the third is the temperature inlet to the degasser. The parameters chosen for the investigation of the three affected streams fell into two categories: sulfur production capacity and environmental impact. The effect of lowering the combustion air temperature inlet to the reaction furnace is investigated in four items: the effect on thermal reactor outlet temperature, the effect on thermal reactor outlet sulfur conversion, the effect on thermal reactor outlet impurities (COS and CS_2_), and the effect on ammonia outlet from the thermal reactor. The effect of lowering the inlet temperature to catalytic reactor1 is investigated in two items: catalytic reactor1 performance and reactor outlet by products COS and CS_2_ concentration. In the outlet sulfur product H_2_S contamination, decreasing temperature inlet to degassing column is studied.

### Optimization step

The optimization step is critical for determining whether the plant can operate at process temperatures lower than the current operating temperature. Table 4 compares the design temperature, the optimized temperature, and the plant operation temperature. The temperatures of the combustion air to reaction furnace inlet, catalytic reactor1 inlet, and degassing column inlet were all optimized.

### Constraints philosophy

1. The environmental limitations necessary to satisfy environmental regulations must be taken into account in any optimization of SRU units.
2. As CO_2_ is responsible for the generation of the two byproducts COS and CS_2_ in the thermal reactor via the oxidation process, this optimization cannot be done if the feed to the thermal reactor contains a large proportion of CO_2_.
  6 $$\mathrm{CO}_{2} + \mathrm{H}_{2}\mathrm{S} \to \mathrm{COS} + \mathrm{H}_{2}O$$
  7 $$\mathrm{CO}_{2} + 2 \ \mathrm{H}_{2}\mathrm{S} \to \mathrm{CS}_{2} + 2 \ \mathrm{H}_{2}\mathrm{O}$$

Ibrahim et al. explained in detail the constraints used for the optimized case from both the exergy and cost optimization perspectives in the article “Energy and exergy studies of a sulfur recovery unit in normal and optimized cases: A real starting up plant”. The current article describes the environmental perspective for the SRU study (Ibrahim et al., 2022).

## Case study and results

The case study results involve the following items: (plant performance guarantee test run checks, process sensitivity analysis and optimization, effect of decreasing combustion air temperature inlet to reaction furnace, effect of decreasing temperature inlet to catalytic reactor1, effect decreasing temperature inlet to degassing column, effect of (H_2_S/SO_2_) ratio on catalytic reactors sulfur conversion, and finally the cost saving from the study).

### Process description and data [13, 14]

**Fig. 3:**
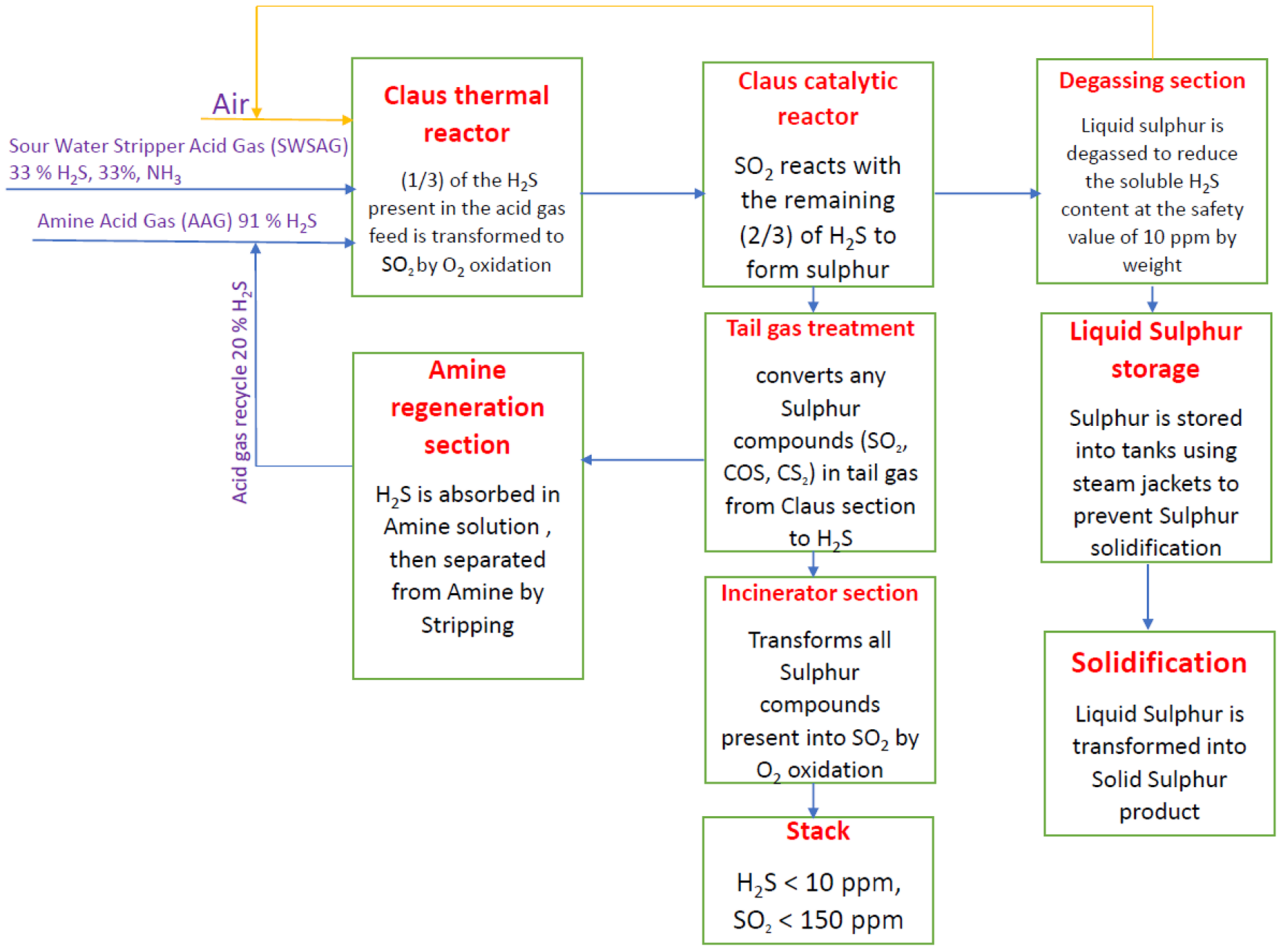
Detailed block diagram for the plant

Sulfur is recovered from amine acid gas and sour water stripper acid gas by the sulfur recovery unit (SRU) and tail gas treatment unit (TGTU). Details are added to plant sections (solidification package, liquid sulfur storage, amine regeneration section) as Fig. 3 describes the plant.

The feed to the sulfur recovery unit is made up of amine acid gas feed with a high percentage of H2S and sour water stripper acid gas with a high percentage of H_2_S and NH_3_. Table 1 shows SRU feed characteristics. In the amine acid gas stream, the H_2_S content is 0.912 mol fraction and 0.327 in the sour water stripper acid gas. The sour water stripper acid gas contains 0.334 mol fraction of ammonia, while the amine acid gas stream contains no ammonia.

**Table 1:** The characteristics of sulfur recovery unit feed

**Stream description**		**Amine acid gas**	**Sour water stripper acid gas**
**Property**	**Unit**	**Industrial**	**Industrial**
Temperature	°C	55	92
Pressure	Kg/cm^2^ g	0.75	0.77
Flow	kg/h	11,975	3674
**Component**		**Mole fraction**	
H_2_		0.003	0.000
H_2_O		0.083	0.339
CO		-	-
N_2_		-	-
O2		-	-
CO_2_		-	-
H_2_S		0.912	0.334
SO_2_		-	-
COS		-	-
CS_2_		-	-
CH_4_		0.001	0.000
C_2_H_6_		-	-
C_3_H_8_		-	-
C_4_H_10_		-	-
C_5_H_12_		-	-
C_6_H_14_		-	-
S_1_		-	-
S_2_		-	-
S_3_		-	-
S_4_		-	-
S_5_		-	-
S_6_		-	-
S_7_		-	-
S_8_		-	-
S liq		-	-
NH_3_		-	0.327

### Process simulation and validation results [13]

**Fig. 4:**
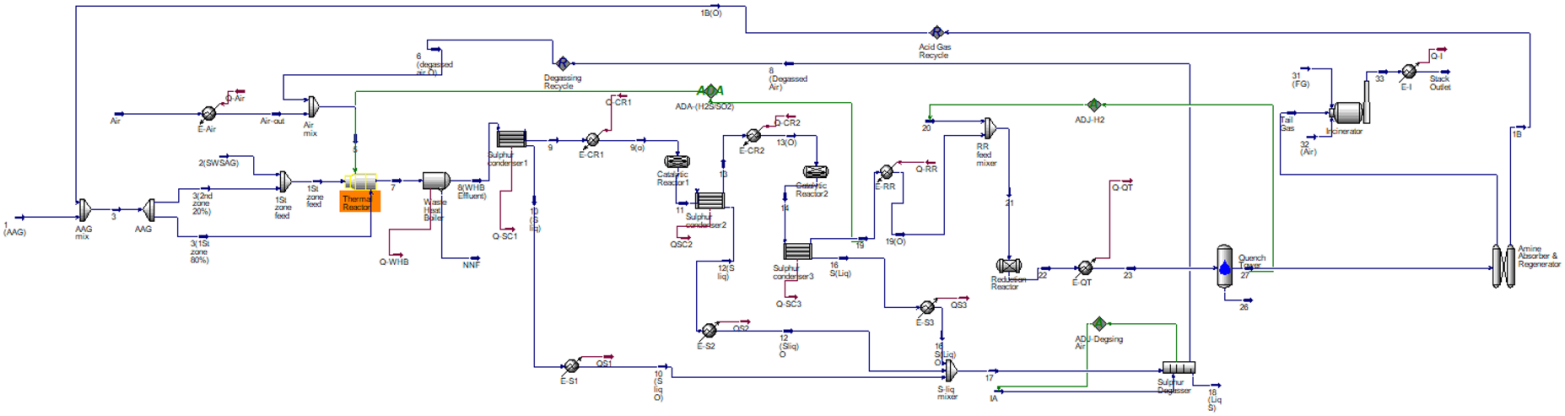
Process simulation (thermal, catalytic, tail gas treatment, degasser, and incinerator sections)

The whole plant is simulated using HYSYS V.11 with Sulsim software using the plant industrial data. Figure 4 shows the PDF from simulation consisting of thermal, catalytic, tail gas treatment, degasser, and incinerator sections).

Table 2 shows the validation results of process streams, covering operating conditions and components compositions. Table 3, in addition, shows reactors validation results. From the overall recovery efficiency point of view, a simulated value of 99.96% is achieved versus 99.90% industrially. Product and flue gas streams comparison between industrial and simulation results is shown in Table 2 and shows accurate results. The percentage error deviation between based case and industrial data is low in plant conditions and composition.

**Table 2:** Comparison between industrial and simulation data for product stream and flue gas streams

Stream description		Liquid sulfur product			Flue gas to stack		
Property	Unit	Industrial	Simulation	% error	Industrial	Simulation	% error
Temperature	°C	135	135	-	652	652	-
Pressure	kg/cm^2^ g	0.01	0.01	-	0.01	0.01	-
Flow	kg/h	12,430	12,438	0.1	41,283	43,002	4.2
**Component**		Mole fraction					
H_2_		0.000	0.000	0.0	0.012	0.011	8.3
H_2_O		0.000	0.000	0.0	0.116	0.120	3.6
CO		0.000	0.000	0.0	0.000	0.000	0.0
N_2_		0.000	0.000	0.0	0.828	0.819	1.0
O_2_		0.000	0.000	0.0	0.020	0.020	1.4
CO_2_		0.000	0.000	0.0	0.025	0.023	5.3
H_2_S		0.000	0.000	0.0	0.000	0.000	0.0
SO_2_		0.000	0.000	0.0	0.000	0.000	0.0
COS		0.000	0.000	0.0	0.000	0.000	0.0
CS_2_		0.000	0.000	0.0	0.000	0.000	0.0
CH_4_		0.000	0.000	0.0	0.000	0.000	0.0
C_2_H_6_		0.000	0.000	0.0	0.000	0.000	0.0
C_3_H_8_		0.000	0.000	0.0	0.000	0.000	0.0
C_4_H_10_		0.000	0.000	0.0	0.000	0.000	0.0
C_5_H_12_		0.000	0.000	0.0	0.000	0.000	0.0
C_6_H_14_		0.000	0.000	0.0	0.000	0.000	0.0
S_1_		0.000	0.000	0.0	0.000	0.000	0.0
S_2_		0.000	0.000	0.0	0.000	0.000	0.0
S_3_		0.000	0.000	0.0	0.000	0.000	0.0
S_4_		0.000	0.000	0.0	0.000	0.000	0.0
S_5_		0.000	0.000	0.0	0.000	0.000	0.0
S_6_		0.000	0.000	0.0	0.000	0.000	0.0
S_7_		0.000	0.000	0.0	0.000	0.000	0.0
S_8_		0.000	0.000	0.0	0.000	0.000	0.0
S liq		1.000	1.000	0.0	0.000	0.000	0.0
NH_3_		0.000	0.000	0.0	0.000	0.000	0.0

**Table 3:** Comparison between industrial and simulation data for reactors outlet temperature

Reactor	Unit	Industrial	Simulation
Thermal reactor outlet temperature	°C	1353	1349
Catalytic reactor1 outlet temperature	°C	298	299.6
Catalytic reactor2 outlet temperature	°C	215	218.9

The comparison between industrial and simulation data for reactors outlet temperature is shown in Table 3. Deviation is very low between both cases, thermal reactor outlet temperature deviation is 4 °C, catalytic reactor1 outlet temperature deviation is 1.6 °C, and catalytic reactor2 outlet temperature deviation is 3.9 °C.

### Plant performance guarantee test run checks

The plant’s official production date is 2020. During December 2019, the SRU underwent a performance acceptance test run to compare the unit’s performance to the licensor’s process performance guarantees. The test run lasted three consecutive days (72 h).

Sulfur tank level transmitters were used by the contractor to calculate the production rate. With the same acid gas feeds flow rates during the test run, the production rate was found to be 1 ton/h lower than the production rate calculated by the availability level transmitters of the sulfur product tanks. This revealed that the test run had failed, so the level transmitters were recalibrated, and the test was repeated in January 2020.

### Process sensitivity analysis and optimization

Three exchangers in the plant encountered actual problems: process side streams were heated by steam, steam valves were opened to 100% without achieving the design values of the process streams, and the decision was made to modify equipment, such as purchasing steam valves of different sizes, or to study the availability to work in the process under the same conditions.

A HYSYS simulation study concluded that working on process side streams at lower temperatures was feasible without affecting overall process recovery.

Table 4 shows plant conditions and optimized conditions versus design. The optimized temperatures are combustion air to reaction furnace inlet temperature (220 °C optimized versus 240 °C design), catalytic reactor1 inlet temperature (220 °C optimized versus 240 °C design), and degassing column inlet temperature (125 °C optimized versus 135 °C design).

**Table 4:** A comparison of the design temperature, the optimized temperature, and the plant operation temperature

	Unit	Design	Plant conditions	Optimized conditions
Combustion air to reaction furnace inlet temperature	°C	240	227	220
Catalytic reactor1 inlet temperature	°C	240	235	220
Degassing column inlet temperature	°C	135	127	125

Lowering the temperature of these exchangers may have a negative impact on sulfur production or the environment, as well as the formation of undesirable ammonium salts, which may damage some equipment in the process.

#### Effect of decreasing combustion air temperature inlet to reaction furnace

The study includes parameters affecting reaction furnace (thermal reactor). Table 5 shows all of them. The parameters are the outlet temperature from thermal reactor, the outlet sulfur conversion, the outlet impurities (COS and CS2) concentration, and the outlet ammonia concentration.

**Table 5:** Effect of decreasing combustion air temperature on thermal reactor

Combustion air inlet temperature	Outlet temperature	Sulfur outlet conversion (%)	COS outlet ppm-mol	CS2 outlet ppm-mol	Ammonia outlet mole fraction
220	1340	69.06	7.30	8.03	0.00
225	1342	69.07	7.02	7.79	0.00
230	1344	69.07	6.73	7.56	0.00
235	1347	69.08	6.45	7.34	0.00
240	1349	69.08	6.16	7.12	0.00

##### Effect on thermal reactor outlet temperature

**Fig. 5:**
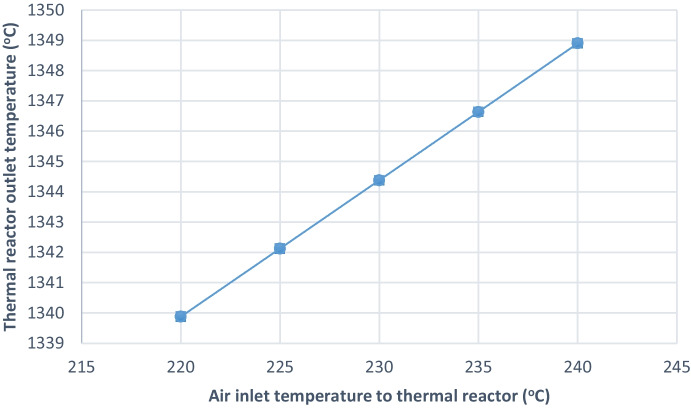
Effect of decreasing air inlet temperature to thermal reactor on outlet temperature from thermal reactor

Decreasing the combustion air inlet temperature from (240 to 220 °C) decreases the thermal reactor outlet temperature from (1349 to 1340 °C) that may lead to bad effects in the process and environmentally due to the formation of more COS and CS_2_ as shown in Fig. 5 and Table 5. COS and CS_2_ are hydrolyzed by (Eqs. 3 and 4) to produce H_2_S in catalytic reactor1, and in reduction reactor, more COS and CS_2_ concentration may affect the ability of catalytic reactor1 and reduction reactor to perform the hydrolysis reaction leading to stack exit with COS and CS_2_.

##### Effect on thermal reactor outlet sulfur conversion

**Fig. 6:**
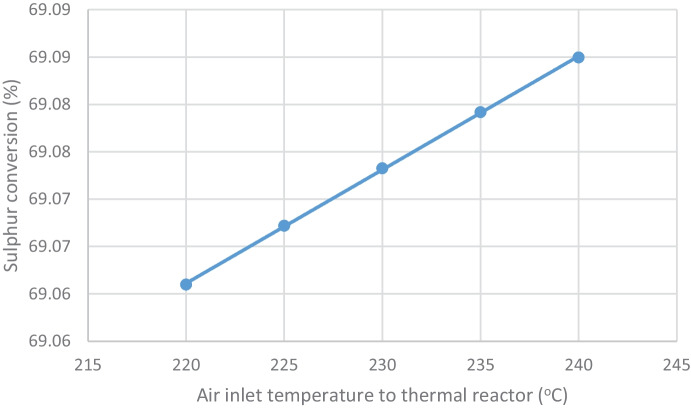
Effect of decreasing air inlet temperature to thermal reactor on thermal reactor sulfur conversion

Outlet sulfur conversion from thermal reactor approximately remains constant (ranges from 69.06 to 69.08%) as shown in Table 5 and Fig. 6. This is a good indication that catalytic reactor1 and catalytic reactor2 performing also sulfur conversion will not be affected by high load.

##### Effects on outlet impurities (COS and CS_2_)

**Fig. 7:**
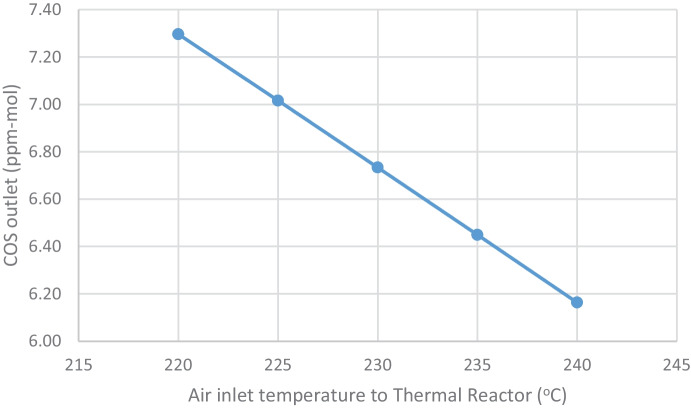
Effect of decreasing air inlet temperature to thermal reactor on COS outlet from thermal reactor

COS increases from (6.16 to 7.3 ppm-mol) by decreasing the combustion air inlet temperature from (240 to 220 °C) as shown in Table 5 and Fig. 7; this increase may lead to bad effects in the process and environmentally.

**Fig. 8:**
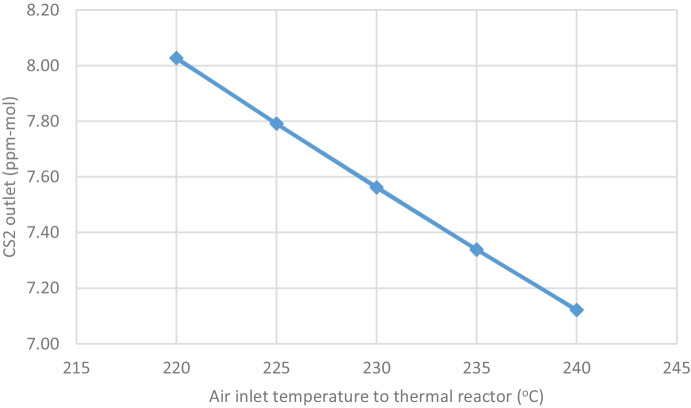
Effect of decreasing air inlet temperature to thermal reactor on CS_2_ outlet from thermal reactor

Table 5 and Fig. 8 show that CS_2_ increases from (7.12 to 8.03 ppm-mol) by decreasing the combustion air inlet temperature.

##### Effect on ammonia outlet

A rapid drop in reaction furnace temperature has a negative impact on ammonia oxidation. The high reaction furnace temperature ensures that the process’s high ammonia content is destroyed. Table 5 shows that there is no ammonia output from the thermal reactor. It is necessary to ensure complete ammonia conversion to N2 and H2O in the reaction furnace to avoid the formation of ammonium salts, which can cause serious problems in the process.

#### Environmental and production effect of decreasing air inlet temperature to thermal reactor

It is required to study how the process handled the side effects of decreasing combustion air inlet temperature on the production and the environment.

##### Environmental effect

As a major goal of the sulfur recovery unit, it is critical to test the environmental impact of decreasing temperature. Lowering the temperature of the reaction furnace increases the amount of byproducts and impurities that exit the reaction furnace. The main significant impurities are COS and CS_2_, and catalytic reactor1 performs the hydrolysis reaction of these components (refer to the Introduction). The study shows that there is a slight increase in the outlet ppm of both compounds from the reaction furnace, but this slight increase is handled in the reduction reactor, which can also perform the hydrolysis reaction. In all cases, the outlet composition mole fraction of COS and CS_2_ from the reduction reactor is 0.

Table 6 displays the sulfur compounds emitted by the reduction reactor and stack. The incinerator’s flue gas outlet does not contain any (H_2_S, SO_2_, S_x_, COS, or CS_2_ sulfur compounds), making the study environmentally successful. Although other SRU researchers’ studies discussed increasing combustion air temperature, I believe the success of the study was due to the high H2S feed concentration (0.912 in amine acid gas and 0.334 in sour water stripper acid gas) that guaranteed furnace flame stability as well as the low concentration of CO_2_ inlet to the furnace.

**Table 6:** Effect of decreasing combustion air inlet temperature on sulfur compounds outlet from the stack, COS, and CS_2_ outlet from reduction reactor

Combustion air inlet temperature (°C)	H_2_S mole fraction in flue gas from stack	SO_2_ mole fraction in flue gas from stack	S_x_ sulfur compounds mole fraction in flue gas from stack	COS mole fraction outlet from reduction reactor	CS_2_ mole fraction outlet from reduction reactor
220	0.00	0.00	0.00	0.00	0.00
225	0.00	0.00	0.00	0.00	0.00
230	0.00	0.00	0.00	0.00	0.00
235	0.00	0.00	0.00	0.00	0.00
240	0.00	0.00	0.00	0.00	0.00

##### Sulfur production

Sulfur production remains constant without any losses, with the goal of keeping sulfur conversion from thermal reactors roughly constant (ranges from 69.08 to 69.06%), as shown in Table 5 and Fig. 6. Any slight decrease in thermal reactor conversion is easily handled by catalytic reactor1 or catalytic reactor2, which also perform the Claus reaction (refer to the Introduction). Table 7 shows that the liquid sulfur product mass flow remains constant at 12,438 kg/h, the liquid sulfur mass fraction remains constant at 1, and the H_2_S ppm-weight in the liquid sulfur product remains constant at 0.

**Table 7:** Sulfur production parameters

Combustion air inlet temperature (°C)	Liquid sulfur product mass flow (kg/h)	Liquid sulfur product mass fraction	H_2_S ppm-weight in liquid sulfur product
220	12,438	1.00	0.00
225	12,438	1.00	0.00
230	12,438	1.00	0.00
235	12,438	1.00	0.00
240	12,438	1.00	0.00

#### Decreasing temperature inlet to catalytic reactor1

The temperature of the catalytic reactor was reduced from (240 to 220 °C). The study considered the effect of this decrease on the performance of the catalytic reactor first, then the effect on sulfur production, and the environment. The hydrolysis reaction efficiency, sulfur conversion efficiency, and mass flow rates of COS and CS_2_ (kg/h) outlet from catalytic reactor1 are important parameters to investigate. Although the efficiency of the hydrolysis reactions decreases slightly, the impurities exiting the reduction reactor remain constant in the absence of COS and CS_2_, as the reduction reactor can also handle hydrolysis reactions.

##### Catalytic reactor1 performance

**Fig. 9:**
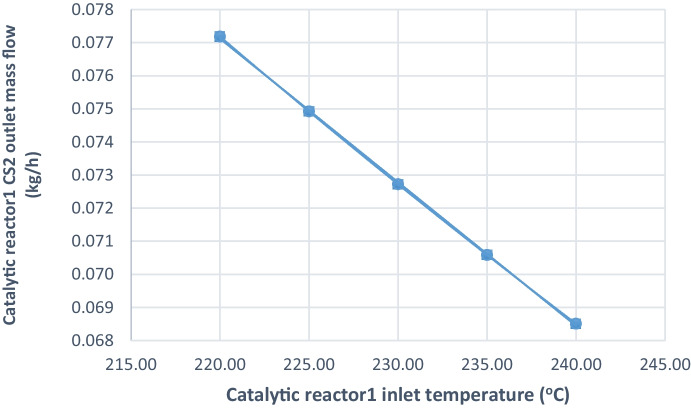
Effect of decreasing inlet temperature of catalytic reactor1 on CS2 outlet

The performance parameters of the catalytic reactor1 are shown in Table 8; COS and CS_2_ hydrolysis percentages are approximately constant, sulfur conversion efficiency is approximately constant, the outlet mass flow rate of COS is approximately constant, and a slight increase in mass flow rate of CS_2_ outlet is observed with decreasing temperature (0.069 to 0.077) kg/h as shown in Fig. 9. The hydrolysis reactions in the reduction reactor handle this minor increase (Eqs. 3 and 4).

**Table 8:** Effect of decreasing inlet temperature on catalytic reactor1 performance

Catalytic reactor1 inlet temperature (°C)	COS hydrolysis result %	CS_2_ hydrolysis result %	Catalytic reactor1 sulfur conversion efficiency	Catalytic reactor1 COS outlet mass flow (kg/h)	Catalytic reactor1 CS_2_ outlet mass flow (kg/h)
220.00	99.04	92.69	71.03	0.069	0.077
225.00	99.04	92.69	71.03	0.069	0.075
230.00	99.04	92.69	71.03	0.069	0.073
235.00	99.04	92.69	71.03	0.069	0.071
240.00	99.05	92.69	71.03	0.070	0.069

##### Reduction reactor outlet COS and CS2

Table 6 shows that outlet from reduction reactor does not contain COS and CS_2_.

#### Environmental and production study of decreasing inlet temperature of catalytic reactor1

As a major goal of the sulfur recovery unit, it is critical to test the environmental impact of decreasing temperature. Lowering the temperature of the reaction furnace increases the amount of byproducts and impurities that exit the reaction furnace. The main significant impurities are COS and CS_2_, and catalytic reactor1 performs the hydrolysis reaction of these components (refer to the Introduction). The study shows that there is a slight increase in the outlet ppm of both compounds from the reaction furnace, but this slight increase is handled in the reduction reactor, which can also perform the hydrolysis reaction. In all cases, the outlet composition mole fraction of COS and CS_2_ from the reduction reactor is 0.

Table 6 shows the outlet sulfur compounds from the reduction reactor and stack. The incinerator’s flue gas outlet does not contain any (H2S, SO2, Sx, COS, or CS2 sulfur compounds), making the study environmentally successful. The authors believe that the study’s success, even though other SRU researchers’ studies discussed increasing combustion air temperature, is due to the high H2S feed concentration (0.912 in amine acid gas and 0.334 in sour water stripper acid gas) that ensured furnace flame stability, preventing a high increase in COS and CS2 by-products out of the reaction furnace. In addition, the production results remain the same as shown in Table 7.

#### Decreasing temperature inlet to degassing column

The temperature of the instrument’s air inlet dropped from 135 to 125 °C. The investigation focuses on the H_2_S contamination of the outlet sulfur product and the mass fraction of sulfur liquid product. Table 9 shows that by lowering the temperature, the product sulfur is not contaminated with H_2_S. The product is then safely transferred to the liquid sulfur tank and the solidification unit.

**Table 9:** Effect of decreasing degassing column inlet temperature on H_2_S contamination in sulfur product

Degassing column air inlet temperature (°C)	H_2_S ppm-weight in liquid sulfur product	Liquid sulfur product mass fraction
125.00	0.00	1.00
127.50	0.00	1.00
130.00	0.00	1.00
132.50	0.00	1.00
135.00	0.00	1.00

#### Effect of (H_2_S/SO_2_) ratio on catalytic reactors sulfur conversion

**Fig. 10:**
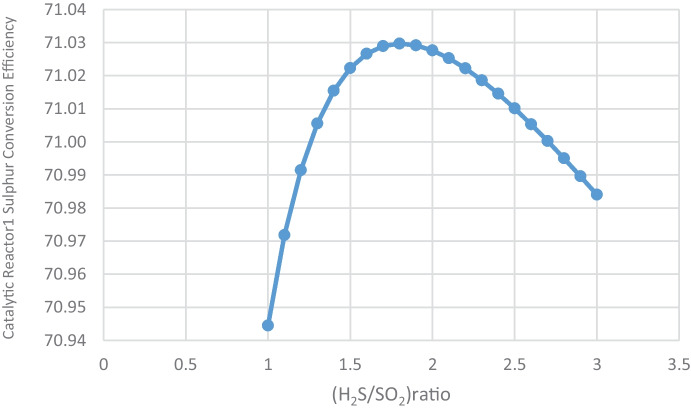
Effect of (H_2_S/SO_2_) ratio on catalytic reactor1 sulfur conversion efficiency

**Fig. 11:**
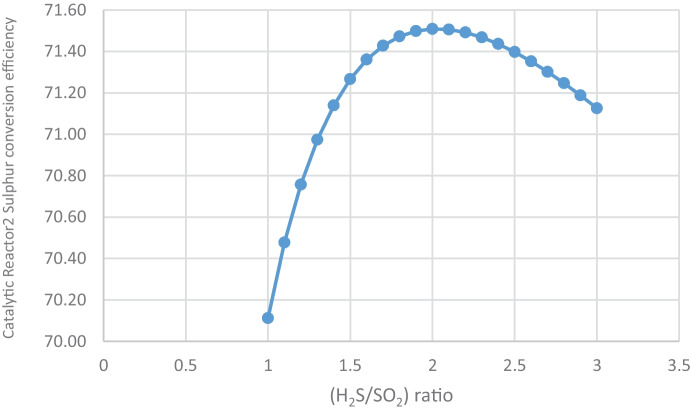
Effect of (H_2_S/SO_2_) ratio on catalytic reactor2 sulfur conversion efficiency

For sulfur conversion, the optimal (H_2_S/SO_2_) ratio is 2. Table 10 and Figs. 10 and 11 show that catalytic reactor1 conversion efficiency is at a maximum of 71.03% at 2 and begins to decrease after 2.2 ratio, while catalytic reactor2 conversion efficiency is at a maximum of 71.51% at 2 and begins to decrease after 2.2 ratio.

**Table 10:** (H_2_S/SO_2_) ratio and sulfur conversion efficiency, sulfur production

(H_2_S/SO_2_) ratio	Catalytic reactor1 conversion efficiency	Catalytic reactor2 conversion efficiency
1	70.94	70.11
1.10	70.97	70.48
1.20	70.99	70.76
1.30	71.01	70.97
1.40	71.02	71.14
1.50	71.02	71.27
1.6	71.03	71.36
1.7	71.03	71.43
1.8	71.03	71.47
1.9	71.03	71.50
2	71.03	71.51
2.1	71.03	71.51
2.2	71.02	71.49
2.3	71.02	71.47
2.4	71.01	71.44
2.5	71.01	71.40
2.6	71.01	71.35
2.7	71.00	71.30
2.8	71.00	71.25
2.9	70.99	71.19
3	70.98	71.13

### Cost saving

The total cost savings calculated from the three exchangers is 69,247.03 $ per year. The article “Energy and exergy studies of a sulfur recovery unit in normal and optimized cases: A real starting up plant” describes the cost savings in detail (Ibrahim et al., 2022).

## Summary and conclusions

In this research, a refinery plant for sulfur recovery in the Middle East starting official production in 2020 is simulated. The actual problems of the test period have been studied. The simulation was then used for optimization and to assure the performance test guarantee calculations done by the contractor. The validation has been done by comparing industrial data with simulation results, and it proved the ability of the simulation to handle different situations. The results are confirmed by actual plant situation and SRU vendor reply. The simulation showed an inaccurate production capacity calculated using plant instrument devices (around 1 ton/h more capacity than the simulation that have an overall recovery efficiency of 99.96%); instrumentation devices were calibrated, and the test run was repeated again. Three exchangers in the plant faced actual problems: process side streams are heated by steam, steam valves are opened by 100% without achieving the design values of the process streams, and the decision is to modify equipment as purchasing new size steam valves, or to study the availability to work in the process with the same conditions. The simulation study concluded the feasibility to work on the process side streams with lower temperatures without affecting the overall recovery of the process. The study succeed environmentally, although other SRU researchers’ studies talked about increasing combustion air temperature and catalytic reactor1 temperature. The purpose is the high H_2_S feed concentration (0.912 in amine acid gas and 0.334 in sour water stripper acid gas) that guaranteed furnace flame stability. The study recommends working lower than design temperatures in similar cases having high H_2_S feed concentration for cost saving. The combustion air inlet temperature of reaction furnace decreased from 240 to 220 °C without affecting sulfur production or environmental limitations. Outlet sulfur conversion from thermal reactor approximately remains constant (ranges from 69.08 to 69.06%). Side effects of decreasing the combustion air inlet temperature are decreasing the thermal reactor outlet temperature from (1349 to 1340 °C), increasing COS side product outlet from thermal reactor from (6.16 to 7.3 ppm-mol), and increasing CS_2_ side product from (7.12 to 8.03 ppm-mol). Although the thermal reactor outlet temperature decreased, no outlet ammonia from the thermal reactor. Although outlet COS and CS_2_ from Thermal reactor increased, no COS and CS_2_ from reduction reactor due to its ability to handle COS and CS_2_ hydrolysis reaction. In overall, the production rate remains the same 12,438 kg/h, and no acidic gas emission from the incinerator stack. The catalytic reactor1 inlet temperature decreased from 240 to 220 °C without affecting sulfur production or environmental limitations. COS and CS_2_ hydrolysis percentage approximately remains constant, sulfur conversion efficiency remains constant, the outlet mass flow rate of COS approximately remains constant, and a little increase of mass flow rate of CS^2^ outlet is observed (0.069 to 0.077) kg/h that is handled in the reduction reactor. The degassing column instrument air inlet temperature decreased from (135 to 125 °C) without contamination sulfur product with H2S. The study shows that the optimum H_2_S/SO_2_ ratio for sulfur conversion in catalytic reactor1 and catalytic reactor2 is 2. Maximum sulfur conversion efficiency of catalytic reactor1 is 71.03% at (H2S/SO2) ratio of 2. Maximum sulfur conversion efficiency of catalytic reactor1 is 71.51% at (H_2_S/SO_2_) ratio of 2. The total amount of steam saved by the three exchangers is 1040.12 kg/h. The average cost of steam is $7.6 per ton. The total calculated cost savings from the three exchangers is 69247.03 $ per year.

## Data Availability

All the data required are included in the manuscript.

## Abbreviations

AAG: Amine acid gas
ADA: Air demand analyzer
ARU: Amine regeneration unit
CFD: Computational fluid dynamics
DEA: Diethanolamine
MDEA: Methyl diethanolamine
PTG: Performance test guarantee
SRE: Sulfur recovery efficiency
SRU: Sulfur recovery unit
SWS: Sour water stripping
SWSAG: Sour water stripped acid gas
TGT: Tail gas treatment section
TGTU: Tail gas treatment
WHB: Waste heat boiler

## References

[CR1] Abdoli P, Hosseini SA, Mujeebu MA (2019). Effect of preheating inlet air and acid gas on the performance of sulfur recovery unit—CFD simulation and validation. Forschung Im Ingenieurwesen.

[CR2] Abdolahi-Mansoorkhani H, Seddighi S (2019). H2S and CO2 capture from gaseous fuels using nanoparticle membrane. Energy.

[CR3] Aghel B, Sahraie & S., Heidaryan, E.,  (2019). Carbon dioxide desorption from aqueous solutions of monoethanolamine and diethanolamine in a microchannel reactor. Separation and Purification Technology.

[CR4] Amini J, Davoodi A, Jafari H (2018). Analysis of internal cracks in type 304 austenitic stainless steel cladding wall of regenerator column in amine treating unit. Engineering Failure Analysis.

[CR5] Concepción E, I., Moreau, A., Martín, M., C., Vega-Maza, D., Segovia, J., J.,  (2020). Density and viscosity of aqueous solutions of methyldiethanolamine (MDEA) + diethanolamine (DEA) at high pressures. The Journal of Chemical Thermodynamics.

[CR6] Dardor D, Janson A, AlShamari E, Adham S, Minier-Matar J (2019). The effect of hydrogen sulfide oxidation with ultraviolet light and aeration. Separation and Purification Technology.

[CR7] Gai H, Chen S, Lin K, Zhang X, Wang C, Xiao M, Huang T, Song H (2020). Conceptual design of energy-saving stripping process for industrial sour water. Chinese Journal of Chemical Engineering.

[CR8] Ghahraloud H, Farsi M, Rahimpour MR (2017). Modeling and optimization of an industrial Claus process: Thermal and catalytic section. Journal of the Taiwan Institute of Chemical Engineers.

[CR9] Hassan-Beck H, Firmansyah T, Suleiman M, I., Matsumoto, T. & AL-Musharfy, M., Chaudry, A., Abdur-Rakiba, M.  (2019). Failure analysis of an oil refinery sour water stripper overhead piping loop: Assessment and mitigation of erosion problems. Engineering Failure Analysis.

[CR10] Hosseini SM, Alizadeh R, Alizadehdakhel A, Behjat Y, Nooriasl P (2019). Enhancement of gas distribution uniformity in a Claus process catalytic reactor using computational fluid dynamics. Chemical Engineering and Processing-Process Intensification.

[CR11] Hashemi M, Pourfayaz F, Mehrpooya M (2019). Energy, exergy, exergoeconomic and sensitivity analyses of modified Claus process in a gas refinery sulfur recovery unit. Journal of Cleaner Production.

[CR12] Ibrahim A, Y., Ashour, F., H., Gadallah, M., A.  (2021). Exergy study of amine scrubber unit of a sulphur recovery plant using methyl diethanolamine: A real starting up plant. Petroleum and Coal.

[CR13] Ibrahim A, Y., Ashour, F., H., Gadallah, M., A.  (2021). Exergy study of amine regeneration unit using diethanolamine in a refinery plant: A real start-up plant. Heliyon.

[CR14] Ibrahim A, Y., Ashour, F., H., Gadallah, M.  (2021). Refining plant energy optimization. Alexandria Engineering Journal..

[CR15] Ibrahim, A. Y., Ashour, F. H., & Gadallah, M. A. (2021d). Exergy study of amine regeneration unit for diethanolamine used in refining gas sweetening: A real start-up plant. *Alexandria Engineering Journal*. 10.1016/j.heliyon.2021.e06241 PMC7900684 33665423

[CR16] Ibrahim, A. Y., Ashour, F. H., & Gadallah, M. A. (2021e). Exergy study of sour water stripper unit of delayed coker unit in a refinery plant: A real start-up plant. *Egyptian Journal of Chemistry*.

[CR17] Ibrahim, A. Y., Ashour, F. H., & Gadallah, M. A. (2021f). Exergy analysis and performance study for sour water stripper units, amine regenerator units and a sulphur recovery unit of a refining plant. *Journal of Engineering and Applied Science.*

[CR18] Ibrahim, A. Y. (2021a). Performance assessment of a sulphur recovery unit. *Petroleum and Petrochemical Engineering Journal,* *5*(1).

[CR19] Ibrahim, A. Y. (2021b). Performance monitoring of a sulphur recovery unit: A real startup plant. *Petroleum and Petrochemical Engineering Journal 5*(1).

[CR20] Ibrahim A, Y., Ashour, F., H., Gadallah, M.  (2022). Energy and exergy studies of a sulphur recovery unit in normal and optimized cases: A real starting up plant. Energy Conversion and Management: x..

[CR21] Ibrahim S, Rahman RK, Raj A (2017). Effects of H2O in the feed of sulfur recovery unit on sulfur production and aromatics emission from Claus furnace. Industrial & Engineering Chemistry Research.

[CR22] Kazempour H, Pourfayaz F, Mehrpooya M (2017). Modeling and multi-optimization of thermal section of Claus process based on kinetic model. Journal of Natural Gas Science and Engineering.

[CR23] Khatami A, Heidari Y, Safadoost A, Aleghafouri A, Davoudi M (2016). The activity loss modeling of catalytic reactor of sulfur recovery unit in South Pars Gas Complex (SPGC) 3rd refinery based on percolation theory. Journal of Natural Gas Science and Engineering.

[CR24] Lavery CB, Marrugo-Hernandez JJ, Sui R, Dowling NI, Marriott RA (2019). The effect of methanol in the first catalytic converter of the Claus sulfur recovery unit. Fuel.

[CR25] Mahmoodi B, Hosseini SH, Ahmadi G, Raj A (2017). CFD simulation of reactor furnace of sulfur recovery units by considering kinetics of acid gas (H2S and CO2) destruction. Applied Thermal Engineering.

[CR26] Mehmood A, Alhasani H, Alamoodi N, AlWahedi YF, Ibrahim S, Raj A (2020). An evaluation of kinetic models for the simulation of Claus reaction furnaces in sulfur recovery units under different feed conditions. Journal of Natural Gas Science and Engineering.

[CR27] Minier-Matar J, Janson A, Hussain A, Adham S (2017). Application of membrane contactors to remove hydrogen sulfide from sour. Journal of Membrane Science.

[CR28] Mohamadi-Baghmoleaei M, Hajizadeh A, Zahedizadeh., P., Azin, R., Zendehboudi, S.  (2020). Evaluation of hybridized performance of amine scrubbing plant based on exergy energy, environmental, and economic prospects: A gas sweetening plant case study. Energy.

[CR29] Monnery WD, Hawboldt KA, Pollock AE, Svrcek WY (2001). Ammonia pyrolysis and oxidation in the Claus furnace. Industrial & Engineering Chemistry Research.

[CR30] Pal P, AbuKashabeh A, Al-Asheh S, Banat F (2015). Role of aqueous methyldiethanolamine (MDEA) as solvent in natural gas sweetening unit and process contaminants with probable reaction pathway. Journal of Natural Gas Science and Engineering.

[CR31] Pashaei H, Ghaemi A (2020). CO2 absorption into aqueous diethanolamine solution with nano heavy metal oxide particles using stirrer bubble column: Hydrodynamics and mass transfer. Journal of Environmental Chemical Engineering.

[CR32] Rahman RK, Ibrahim S, Raj A (2019). Multi-objective optimization of sulfur recovery units using a detailed reaction mechanism to reduce energy consumption and destruct feed contaminants. Computers & Chemical Engineering.

[CR33] Rao, N. K., & Haydary, J. (2019). Studies on sulfur recovery plant performance using Aspen HYSYS Sulsim simulations. *Petroleum & Coal, 61*(2).

[CR34] Rostami A, Tavan Y (2019). A survey on exergy, energy and environmental analysis of sulfur recovery unit in case of five intensified configurations. Chemical Papers.

[CR35] Shunji K, Xizhou S, Wenze Y (2020). Investigation of CO2 desorption kinetics in MDEA and MDEA+DEA rich amine solutions with thermo-gravimetric analysis method. International Journal of Greenhouse Gas Control.

[CR36] Sui R, Lavery CB, Li D, Deering CE, Chou N, Dowling NI, Marriott RA (2019). Improving low-temperature CS2 conversion for the Claus process by using La (III)-doped nanofibrous TiO2 xerogel. Applied Catalysis B: Environmental.

[CR37] Wang M, Hariharan S, Shaw R, A. & Hatton, T., A.  (2019). Energetics of electrochemically mediated amine regeneration process for flue gas CO_2_ capture. International Journal of Greenhouse Gas Control.

[CR38] Zahid Z (2019). Techno-economic evaluation and design development of sour water stripping system in the refineries. Journal of Cleaner Production.

[CR39] Zarei S (2020). Exergetic, energetic and life cycle assessments of the modified Claus process. Energy.

[CR40] Zhu M, Sun L, Ou G, Wang K, Wang K, Sun Y (2016). Erosion corrosion failure analysis of the elbow in sour water stripper overhead condensing reflux system. Engineering Failure Analysis.

